# EZH2 expression is a prognostic biomarker in patients with colorectal cancer treated with anti-EGFR therapeutics

**DOI:** 10.18632/oncotarget.14863

**Published:** 2017-01-27

**Authors:** Itaru Yamamoto, Katsuhiko Nosho, Shinichi Kanno, Hisayoshi Igarashi, Hiroyoshi Kurihara, Keisuke Ishigami, Kazuya Ishiguro, Kei Mitsuhashi, Reo Maruyama, Hideyuki Koide, Hiroyuki Okuda, Tadashi Hasegawa, Yasutaka Sukawa, Kenji Okita, Ichiro Takemasa, Hiroyuki Yamamoto, Yasuhisa Shinomura, Hiroshi Nakase

**Affiliations:** ^1^ Department of Gastroenterology and Hepatology, Sapporo Medical University School of Medicine, Sapporo, Japan; ^2^ Department of Molecular Biology, Sapporo Medical University School of Medicine, Sapporo, Japan; ^3^ Department of Oncology, Keiyukai Sapporo Hospital, Sapporo, Japan; ^4^ Department of Surgical Pathology, Sapporo Medical University School of Medicine, Sapporo, Japan; ^5^ Department of Surgery, Surgical Oncology and Science, Sapporo Medical University School of Medicine, Sapporo, Japan; ^6^ Division of Gastroenterology and Hepatology, Department of Internal Medicine, St. Marianna University School of Medicine, Kawasaki, Japan; ^7^ Department of Gastroenterology, Ikeda Municipal Hospital, Ikeda, Japan

**Keywords:** EZH2, microRNA-31, colon cancer, EGFR, CIMP

## Abstract

The polycomb group protein enhancer of zeste homolog 2 (EZH2) is a methyltransferase that suppresses microRNA-31 (miR-31) in various human malignancies including colorectal cancer. We recently suggested that miR-31 regulates the signaling pathway downstream of epidermal growth factor receptor (EGFR) in colorectal cancer. Therefore, we conducted this study for assessing the relationship between EZH2 expression and clinical outcomes in patients with colorectal cancer treated with anti-EGFR therapeutics. We immunohistochemically evaluated EZH2 expression and assessed miR-31 and gene mutations [*KRAS* (codon 61/146), *NRAS* (codon 12/13/61), and *BRAF* (codon 600)] in 109 patients with colorectal cancer harboring *KRAS* (codon 12/13) wild-type. We also evaluated the progression-free survival (PFS) and overall survival (OS). In the result, low EZH2 expression was significantly associated with shorter PFS (log-rank test: *P* = 0.023) and OS (*P* = 0.036) in patients with colorectal cancer. In the low-miR-31-expression group and the *KRAS* (codon 61/146), *NRAS*, and *BRAF* wild-type groups, a significantly shorter PFS (*P* = 0.022, *P* = 0.039, *P* = 0.021, and *P* = 0.036, respectively) was observed in the EZH2 low-expression groups than in the high-expression groups. In the multivariate analysis, low EZH2 expression was associated with a shorter PFS (*P* = 0.046), independent of the mutational status and miR-31. In conclusion, EZH2 expression was associated with survival in patients with colorectal cancer who were treated with anti-EGFR therapeutics. Moreover, low EZH2 expression was independently associated with shorter PFS in patients with cancer, suggesting that EZH2 expression is a useful additional prognostic biomarker for anti-EGFR therapy.

## INTRODUCTION

Monoclonal antibodies against the epidermal growth factor receptor (EGFR) have expanded the spectrum of therapeutic options; they have also improved the clinical outcome of patients with metastatic colorectal cancer [1–6]. The deregulation of the signaling pathways downstream of EGFR, including the RAS/RAF/MEK/ERK pathway, may induce resistance to anti-EGFR therapy. Regarding this resistance, a mutation in *KRAS* codon 61 or 146 has been actively studied as a possible additional predictive biomarker for anti-EGFR therapy [6, 7]. In addition, several studies have suggested that mutations in certain genes (i.e., *NRAS* and *BRAF*) account for this resistance [3–6, 8–16] because they are downstream effectors of the EGFR signaling pathway, similar to the *KRAS* gene. Therefore, there is a need to identify additional biomarkers to more accurate selection of patients for anti-EGFR therapy.

MicroRNAs have been increasingly recognized as useful biomarkers of various human cancers [17–22]. Regarding microRNA in the signaling pathway downstream of EGFR, we recently suggested that microRNA-31 (miR-31)-5p regulates *BRAF* activation in colorectal cancer [23, 24] and that high miR-31-5p is associated with survival in patients with colorectal cancer who underwent surgical treatment and chemotherapy with anti-EGFR antibodies [19].

The polycomb group protein enhancer of zeste homolog 2 (EZH2) is a methyltransferase and the core catalytic element of polycomb repressive complex 2 (PRC2), which plays a critical role in the regulation of cancer initiation, progression, invasion, metastasis, and drug resistance [25–27]. Various oncogenic transcription factors and cancer-associated non-coding RNAs including microRNA regulate EZH2 expression [19, 26, 28–31]. EZH2-mediated histone methylation suppresses miR-31 expression in prostate cancer [29] and adult T-cell leukemia [26]. Regarding colorectal cancer, we recently reported that EZH2 suppresses miR-31 expression by inducing histone H3 lysine 27 trimethylation (H3K27me3) on the miR-31 promoter and that EZH2 inhibition increased miR-31 expression [28].

Thus, accumulating evidence suggests that EZH2 is a useful and additional prognostic biomarker for anti-EGFR therapy in patients with colorectal cancer. Therefore, we conducted this study to assess the relation between EZH2 expression and clinical outcomes in patients with metastatic colorectal cancer treated with anti-EGFR therapeutics.

## RESULTS

### EZH2 expression in 109 patients with colorectal cancer treated with anti-EGFR therapy

The study included 115 patients with metastatic colorectal cancer who were received cetuximab or panitumumab. Immunohistochemistry for EZH2 expression were successfully performed in 109 (95%) colorectal cancers. We excluded six patients because of insufficient EZH2 staining. EZH2 expression scores of 0 (negative), 1 (weak), 2 (moderate), and 3 (strong) were observed in 11%, 21%, 18%, and 50% of the colorectal cancer tissues, respectively (Supplementary Figure 1).

### Association between EZH2 expression and clinical and molecular characteristics in colorectal cancer

Of the 109 patients with colorectal cancer treated with anti-EGFR therapeutics, 50 (46%) received cetuximab and 59 (54%) received panitumumab. The regimen of cetuximab or panitumumab administration corresponded to first-line treatment in 16 (15%) patients, second-line treatment in 17 (16%) patients, and third-line treatment and beyond in 76 (70%) patients.

Regarding miR-31-5p expression, 12 (11%) patients and 97 (89%) patients were classified into the high- and low-expression groups, respectively. The *KRAS* (codon 61/146), *NRAS* mutation (codon 12/13/61), and *BRAF* (codon 600) mutations were detected in 7 (6.4%), 8 (7.3%), and 6 (5.5%) patients, respectively.

Table 1 shows the clinicopathological and molecular features according to the EZH2 expression level. There were no significant associations between EZH2 expression and clinical or molecular features such as gender, age, tumor location, anti-EGFR therapeutics, anti-EGFR therapy line, *BRAF* and *NRAS* mutations. In contrast, a high EZH2 expression was inversely associated with *KRAS* mutation (codon 61/146) (*P* = 0.0039). A high EZH2 expression was inversely associated with miR-31 expression; however, no significant relationship was found between them (*P* = 0.085).

**Table 1:** Clinicopathological or molecular features of 109 colorectal cancer patients who received anti-EGFR therapy

Clinicopathological or molecular feature	Total N	EZH2 expression				*P*
		score 0(negative)	score 1(weak)	score 2(moderate)	score 3 (strong)	
All cases	109	12 (11%)	23 (21%)	20 (18%)	54 (50%)	
Gender						
Male	76 (70%)	7 (58%)	16 (69%)	14 (70%)	39 (72%)	0.84
Female	33 (30%)	5 (42%)	7 (30%)	6 (30%)	15 (28%)	
Age (mean ± SD)	60.5 ± 11.6	61.9 ± 8.2	62.1 ± 13.0	60.4 ± 9.2	59.6 ± 12.4	0.82
Tumor location						
Distal colon (splenic flexure to sigmoid colon) and Rectum	82 (75%)	5 (42%)	17 (74%)	13 (65%)	47 (87%)	0.0082
Proximal colon (cecum to transverse colon)	27 (25%)	7 (58%)	6 (26%)	7 (35%)	7 (13%)	
Anti-EGFR agents						
Cetuximab	50 (46%)	5 (42%)	11 (48%)	9 (45%)	25 (46%)	0.99
Panitumumab	59 (54%)	7 (58%)	12 (52%)	11 (55%)	29 (54%)	
Line of anti-EGFR therapy						
First line	16 (15%)	0 (0%)	2 (8.7%)	3 (15%)	11 (20%)	0.18
Second line	17 (16%)	2 (17%)	6 (26%)	4 (20%)	5 (9.3%)	
Third line and beyond	76 (70%)	10 (83%)	15 (65%)	13 (65%)	38 (70%)	
*BRAF* mutation (codon 600)						
Wild-type	103 (95%)	11 (92%)	22 (96%)	19 (95%)	51 (94%)	0.97
Mutant	6 (5.5%)	1 (8.3%)	1 (4.4%)	1 (5.0%)	3 (5.6%)	
*KRAS* mutation (codon 61/146)						
Wild-type	102 (94%)	9 (75%)	22 (96%)	17 (85%)	54 (100%)	0.0039
Mutant	7 (6.4%)	3 (25%)	1 (4.4%)	3 (15%)	0 (0%)	
*NRAS* mutation (codon 12/13/61)						
Wild-type	101 (93%)	12 (100%)	21 (91%)	19 (95%)	49 (91%)	0.50
Mutant	8 (7.3%)	0 (0%)	2 (8.7%)	1 (5.0%)	5 (9.3%)	
MicroRNA-31-5p expression						
Low-expression	97 (89%)	8 (67%)	21 (91%)	17 (85%)	51 (94%)	0.085
High-expression	12 (11%)	4 (33%)	2 (8.7%)	3 (15%)	3 (5.6%)	

Percentage (%) indicates the proportion of cases with a specific clinicopathological or molecular feature within a given dichotomous category of EZH2 expression by immunohistochemistry. *P* values were calculated by analysis of variance for age and by a chi-square test or Fisher's exact test for all other variables.

EGFR, epidermal growth factor receptor; SD, standard deviation.

### Expression of EZH2 and efficacy of anti-EGFR therapy in *KRAS* (codon12/13) wild-type colorectal cancers

During the follow-up study of the 109 patients with colorectal cancer treated with anti-EGFR therapeutics who were eligible for survival analysis, 64 patients died (all deaths were confirmed to be attributed to colorectal cancer). The median follow-up periods for Progression-free survival (PFS) and overall survival (OS) were 6.1 and 46 months, respectively.

**Figure 1:**
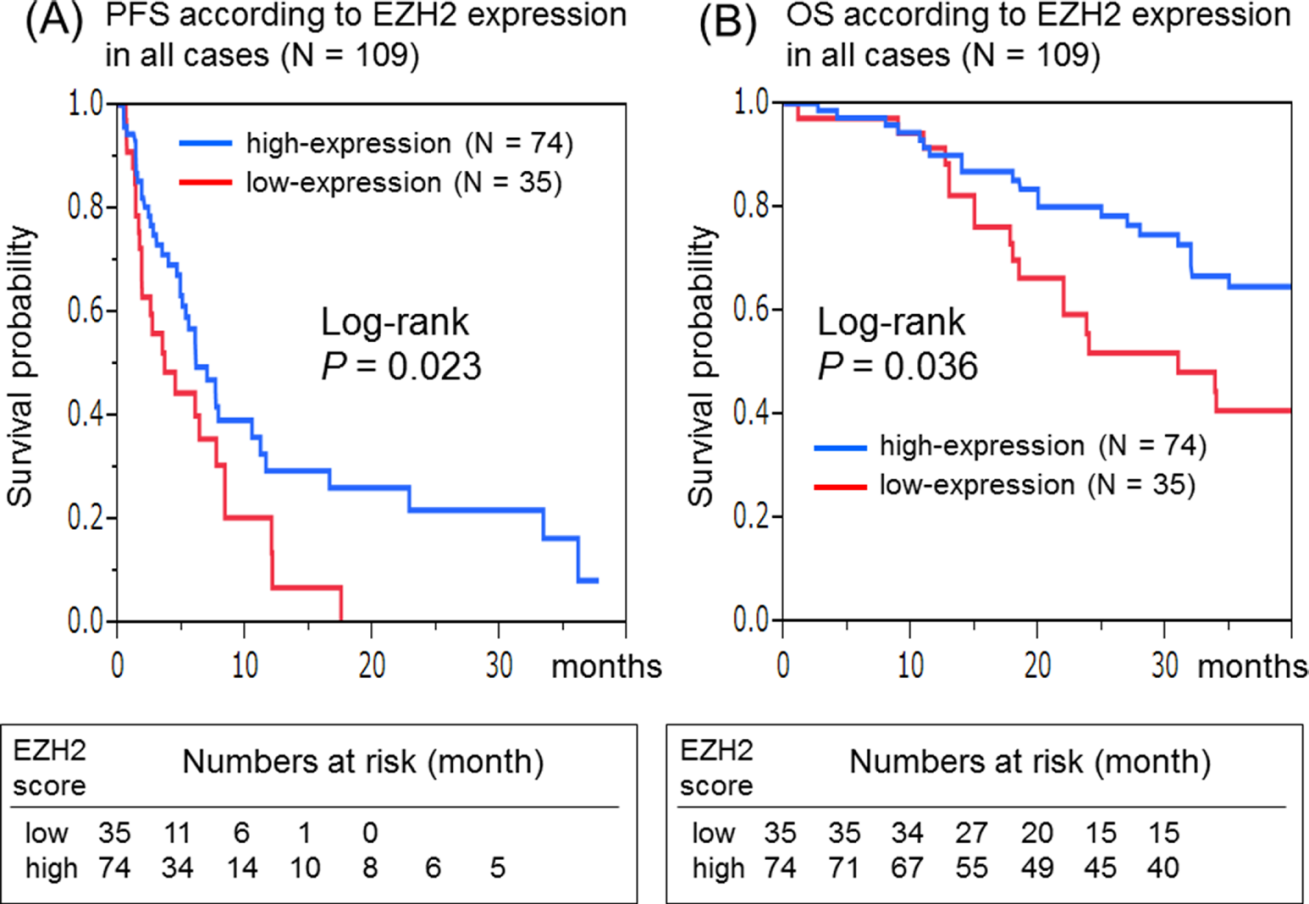
Kaplan–Meier survival curves of patients treated with anti-EGFR therapeutics in all cases (*N* = 109) (**A**) Progression-free survival of the EZH2 low-expression group versus that of the high-expression group. (**B**) Overall survival of the EZH2 low-expression group versus that of the high-expression group.

We made EZH2 into a dichotomous expression variable, defining scores of 2–3 as high-expression and those of 0–1 as low-expression. The median PFS and OS in the EZH2 low-expression group were 3.7 and 31 months, while those in the EZH2 high-expression group were 6.1 and 55 months, respectively. In terms of PFS, a significantly shorter PFS was observed in patients in the low-expression group (log-rank test: *P* = 0.023) than in those in the high-expression group (Figure 1A). Additionally, there was a significant difference in OS (*P* = 0.036) between the EZH2 low-(scores of 0–1) and high- (scores of 2–3) expression groups (Figure 1B). In the univariate Cox regression analysis, a significantly shorter PFS [hazard ratio (HR): 1.79; 95% confidence interval (CI): 1.06–2.98; *P* = 0.029)] and OS (HR: 1.69; 95% CI: 1.02–2.76; *P* = 0.042) were observed in the low-expression group (scores of 0–1) than in the high-expression group (scores of 2–3).

### Expression of EZH2 and efficacy of anti-EGFR therapy according to mutational status in *KRAS* (codon 12/13) wild-type colorectal cancers

**Figure 2:**
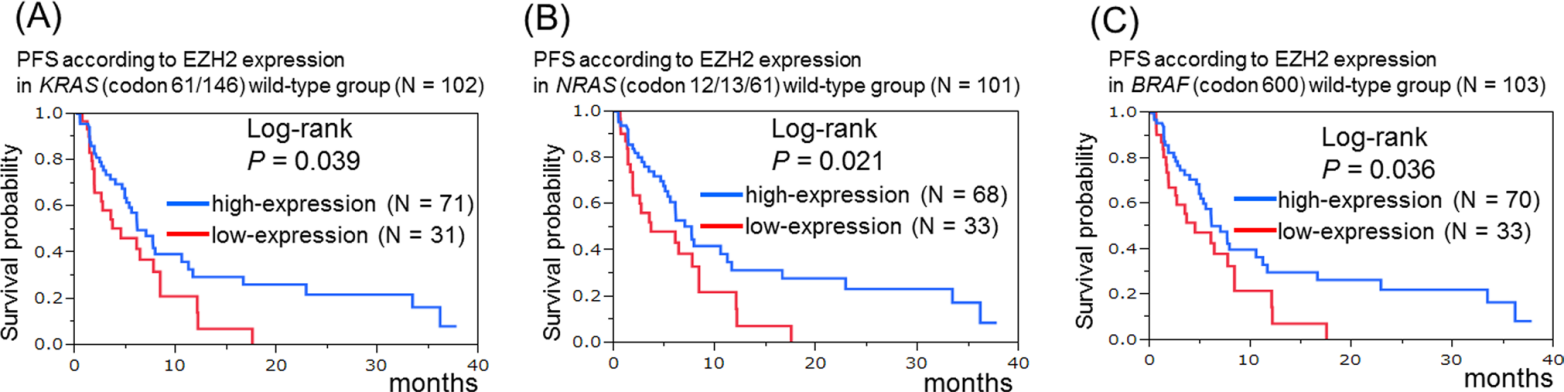
Kaplan–Meier survival curves of patients treated with anti-EGFR therapeutics in wild-type groups of *KRAS* (codon 61/146), *NRAS* (codon 12/13/61), and *BRAF* (codon 600) (**A**) Progression-free survival according to EZH2 expression in the *KRAS* wild-type group (*N* = 102). (**B**) Progression-free survival according to EZH2 expression in the *NRAS* wild-type group (*N* = 101). (**C**) Progression-free survival according to EZH2 expression in the *BRAF* wild-type group (*N* = 103).

We further analyzed the association of EZH2 expression with the efficacy of anti-EGFR therapy in patients with colorectal cancer with no mutations in *KRAS* (codon 61/146), *NRAS* (codon 12/13/61), or *BRAF* (codon 600). In the *KRAS* (codon 61/146), *NRAS*, and *BRAF* wild-type groups, a significantly shorter PFS (log-rank test: *P* = 0.039, *P* = 0.021, and *P* = 0.036, respectively) was observed in the EZH2 low-expression groups (scores of 0–1) than in the high-expression groups (scores of 2–3) in Kaplan–Meier analysis (Figure 2). We also examined the PFS for the group with at least one mutation in *KRAS*, *NRAS*, or *BRAF* and the no-mutation group. As a result, in the no-mutation group, our data indicated a tendency for a significant difference between the EZH2 high- and low-expression groups in Kaplan–Meier analysis (*P* = 0.057).

Regarding OS, significant differences were observed according to EZH2 expression in the *NRAS* (log-rank test: *P* = 0.017), and *BRAF* (log-rank test: *P* = 0.029) wild-type groups; however, no significant difference was observed between the low- and high-expression groups in the *KRAS* wild-type group (codon 61/146; log-rank test: *P* = 0.22) (Supplementary Figure 2).

### Expression of miR-31 and efficacy of anti-EGFR therapy in *KRAS* (codon 12/13) wild-type group colorectal cancers

**Figure 3:**
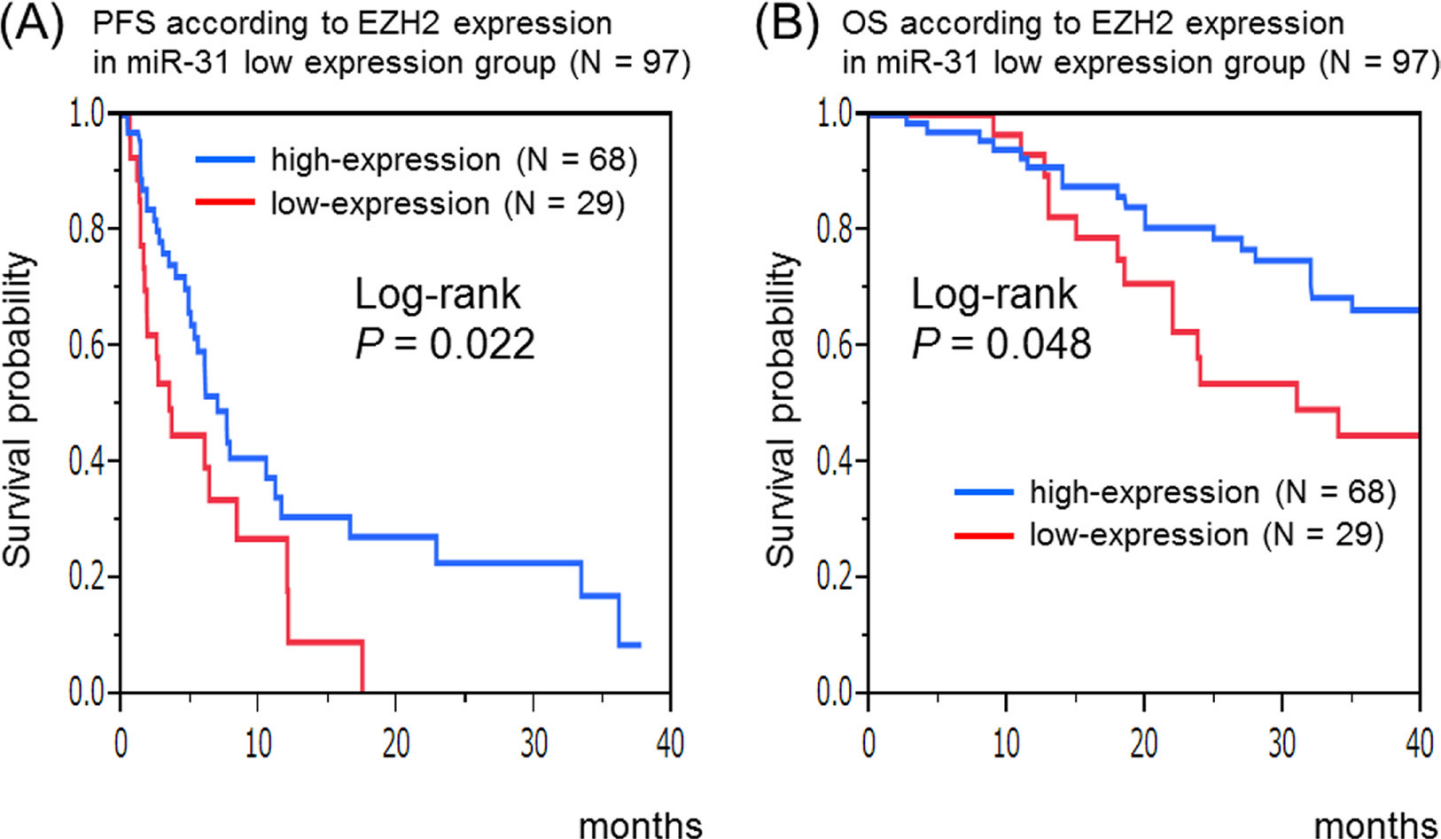
Kaplan–Meier survival curves of patients treated with anti-EGFR therapeutics in the microRNA-31 (miR-31)-5p low-expression group (*N* = 97) (**A**) Progression-free survival according to EZH2 expression in the miR-31 low-expression group. (**B**) Overall survival according to EZH2 expression in the miR-31 low-expression group.

In the miR-31 low-expression group, a significantly shorter PFS (log-rank test: *P* = 0.022) was observed in the EZH2 low-expression group (scores of 0–1) than in the high-expression group (scores of 2–3) in Kaplan–Meier analysis (Figure 3). Similarly, in the miR-31 low-expression group, a significant difference was observed in OS according to EZH2 expression (log-rank test: *P* = 0.048) (Figure 3).

### Multivariate cox regression analysis for PFS and OS according to EZH2 expression in colorectal cancers

In the multivariate Cox regression analysis for PFS, a significantly shorter PFS was observed in the EZH2 low-expression group (scores of 0–1) than in the high-expression group (scores of 2–3) (HR: 1.73; 95% CI: 1.01–2.92; *P* = 0.046) (Table 2). Regarding OS, a poorer prognosis was observed in the EZH2 low-expression group than in the high-expression group, although no significant difference was found between them (HR: 1.56; 95% CI: 0.93−2.58; *P* = 0.093) (data not shown).

**Table 2:** Multivariate Cox regression analysis of colorectal cancer patients treated with anti-EGFR therapy

	Progression-free survival			
	Median(months)	Hazardratio	95%confidence interval	*P*
*NRAS* mutation (codon 12/13/61)(mutated group vs. wild-type group)	2.6 vs. 6.1	3.62	1.32–8.50	0.015
EZH2 expression(low-expression group vs. high-expression group)	3.7 vs. 6.1	1.73	1.01–2.92	0.046
*BRAF* mutation (codon 600)(mutated group vs. wild-type group)	2.0 vs. 6.1	2.97	0.86–7.87	0.080
*KRAS* mutation (codon 61/146)(mutated group vs. wild-type group)	2.5 vs. 6.1	2.93	0.66–9.19	0.14
Tumor location [Proximal colon (cecum to transverse colon) vs.Distal colon (splenic flexure to sigmoid colon) and Rectum]	4.9 vs. 6.1	1.61	0.84–2.96	0.15
MicroRNA-31 expression (high-expression group vs. low-expression group)	4.5 vs. 6.1	1.18	0.55–2.33	0.65

EGFR, epidermal growth factor receptor.

## DISCUSSION

In the current study on patients with colorectal cancer who underwent surgical treatment, we elucidated the association of EZH2 expression, gene mutations, or miR-31 expression in the pathway downstream of EGFR with the efficacy of anti-EGFR therapy. Low EZH2 expression was significantly associated with a shorter PFS and OS in patients with *KRAS* (codon 12/13) wild-type group colorectal cancer treated with anti-EGFR therapeutics. Moreover, low EZH2 expression was associated with shorter PFS, independent of the mutations of *BRAF* (codon 600), *KRAS* (codon 61/146), and *NRAS* (codon 12/13/61) mutations and miR-31 expression. Thus, our data suggest that EZH2 expression is a useful biomarker for anti-EGFR therapy in patients with colorectal cancer.

It is well established that mutations in *KRAS* codon 12 or 13 are predictive of a lack of response to anti-EGFR therapy [1, 2, 32]. Recent studies have demonstrated a correlation between mutation in *KRAS* codons 61 or 146 [3, 6, 7] and resistance to anti-EGFR therapy. Moreover, as additional RAS-activating mutations, *NRAS* mutations have been reported to predict the response in patients with colorectal cancer treated with anti-EGFR therapeutics [3, 6, 33–35]. *BRAF* has also been reported to demonstrate a correlation between mutation and resistance to treatment with anti-EGFR therapy [4, 5, 7, 10, 13, 36]. Regarding the association between microRNA expression and resistance to treatment with anti-EGFR therapy, we recently suggested that high miR-31-5p expression is a useful and additional prognostic biomarker for anti-EGFR therapy [19].

Our current data showed a significantly shorter PFS and OS in the EZH2 low-expression group than in the high-expression group, not only in the wild-type groups of *KRAS* (codon 61/146), *NRAS*, and *BRAF* but also in the miR-31 low-expression group. In addition, the present multivariate analysis including the variable of EZH2 expression stratified by miR-31 and mutations of *BRAF*, *KRAS*, and *NRAS* showed a significantly shorter PFS in the EZH2 low-expression group than in the high-expression group. To our knowledge, this is the first report describing the association between the expression of EZH2 and efficacy of anti-EGFR therapy in colorectal cancer. Unlike our previous report using colorectal cancer (*N* = 301) [28], no significant association was found between EZH2 and miR-31 expression, although there was a trend (*P* = 0.085). The reason for this discrepancy may have been the small sample size in the current study (*N* = 109).

Regarding the association between EZH2 expression and the signaling pathway downstream of EGFR, Riquelme et al. recently reported that oncogenic *KRAS* regulates EZH2 expression in lung cancer and that different amino acid substitutions in *KRAS* mutants differentially modulate EZH2 expression [25]. These results suggest that EZH2 inhibitory strategies can be combined with MEK/ERK-targeted therapies to treat patients with lung cancer. Additionally, they also extended these findings to colon and pancreatic cancers and obtained similar results. Our current study showed a significant correlation between EZH2 expression and *KRAS* (codon 61/146) mutation in colorectal cancers. These findings suggest that oncogenic *KRAS* mutations in different kinds of tumors differentially modulate EZH2 expression through the signaling pathway downstream of EGFR. Further functional analysis is required for clarifying the regulatory role of EZH2 in the signaling pathway downstream of EGFR in colorectal cancer.

There are some limitations to our study. These include the cross-sectional and observational design and the relatively small sample size for prognostic analysis. In addition, all patients underwent resection of the primary lesion of colorectal cancer before chemotherapy. These limitations may have affected the prognostic analysis; OS of patients with colorectal cancer was relatively longer than that obtained in large clinical trials. In contrast, our current study showed that there was a significantly better PFS in colorectal cancers possessing all wild-type copies of *KRAS*, *NRAS*, and *BRAF* than in colorectal cancers with at least one mutation of *KRAS*, *NRAS*, and *BRAF*, although no significant differences in OS were observed between them (data not shown). These findings are almost consistent with those of previous studies [3, 5, 13, 16, 34] and support the validity of our pyrosequencing assay for examining the gene mutations in the pathway downstream of EGFR. Future large and independent studies are necessary for confirming the correlation between low EZH2 expression and unfavorable prognosis in patients with metastatic colorectal cancer who have received anti-EGFR therapy.

In conclusion, we found that EZH2 expression was associated with survival in patients with metastatic colorectal cancer who underwent surgical treatment and chemotherapy with anti-EGFR antibodies. Moreover, low EZH2 expression was associated with a shorter PFS in patients with colorectal cancer, independent of gene mutations in the downstream part of the EGFR pathway and miR-31 expression, suggesting that EZH2 expression is a useful and additional prognostic biomarker for anti-EGFR therapy.

## MATERIALS AND METHODS

### Patients and tissue specimens

We collected formalin-fixed paraffin-embedded (FFPE) tissues of 109 primary tumors of colorectal cancers of patients who underwent surgical treatment and chemotherapy with anti-EGFR antibodies at Sapporo Medical University Hospital or Keiyukai Sapporo Hospital between 1997 and 2014. All patients underwent surgical resection of primary tumor of colorectal cancer before receiving anti-EGFR therapy. To clarify the association between EZH2 expression and survival in patients with metastatic colorectal cancer, we limited the patients who received 5-FU-based adjuvant chemotherapy before receiving anti-EGFR therapy. The primary cancer tissues of all patients were confirmed to be both histologically EGFR-positive and without mutations in *KRAS* codons 12 and 13.

PFS was defined as the time from the beginning of anti-EGFR therapy to progression or death by any cause. Patients who did not meet these criteria were censored at the date of last administration. OS was defined as the time from the diagnosis of colorectal cancer to death by any cause or last follow-up. The patients were followed until death or December 2014, whichever came first. Informed consent was obtained from all patients before specimen collection. This study was approved by the institutional review boards of the participating institutions.

### RNA extraction from FFPE tissues and quantitative reverse transcription-PCR of microRNA

Total RNA was extracted from FFPE tissues using the miRNeasy FFPE Kit (Qiagen, Valencia, CA, USA) [23]. MiR-31-5p expression was analyzed by quantitative reverse transcription-PCR (qRT-PCR) using TaqMan MicroRNA Reverse Transcription Kit (Applied Biosystems, Foster City, CA, USA) and TaqMan microRNA Assays (Applied Biosystems) as previously described [23].

### Pyrosequencing of *BRAF*, *KRAS*, and *NRAS* mutations

Using extracted genomic DNA, PCR and targeted pyrosequencing were performed for *BRAF* (codon 600) [37], *KRAS* (codon 12/13/61/146) [19], and *NRAS* (codon 12/13/61) [33].

### Immunohistochemistry for EZH2 expression

Immunohistochemistry was performed on 5 μm FFPE sections. Sections were autoclave-pretreated in target retrieval solution (Dako Cytomation, Carpinteria, CA, USA). Endogenous peroxidase activity was blocked using 3% hydrogen peroxide, and the sections were incubated with anti-EZH2 antibody as previously described [28]. Five random high-power fields were evaluated per lesion to determine the mean nuclear positivity, which was categorized as follows: score 0 (negative, < 5%), score 1 (weak, 5%–39%), score 2 (moderate, 40%–79%), or score 3 (strong, ≥ 80%).

### Statistical analysis

JMP (version 10) software was used for statistical analysis (SAS Institute, Cary, NC, USA). All *P* values were 2-sided. Univariate analysis was performed to assess clinicopathological and molecular characteristics according to the EZH2 expression level; the chi-square test or Fisher's exact test was used for categorical data, whereas analysis of variance was used to compare the mean patient age.

In survival analysis, the Kaplan–Meier method and log-rank test were used to assess survival time distribution. Cox proportional hazards regression models were used to compute mortality hazard ratio according to the EZH2 expression status. The multivariate Cox model included the variable of EZH2 expression stratified by tumor location (proximal colon vs. distal colon and rectum), the mutations of *BRAF* (codon 600), *KRAS* (codon 61/146), and *NRAS* (codon 12/13/61) (present vs. absent), and miR-31 (high-expression vs. low-expression). A backward elimination was performed with a threshold of *P* = 0.10, to avoid overfitting.

## SUPPLEMENTARY MATERIALS FIGURES AND TABLES



## Abbreviations

CI, confidence interval; EGFR, epidermal growth factor receptor; EZH2, enhancer of zeste homolog 2; FFPE, formalin-fixed, paraffin-embedded; HR, hazard ratio; H3K27me3, histone H3 lysine 27 trimethylation; miR-31, microRNA-31; OS, overall survival; PFS, progression-free survival; SD, standard deviation.
