# Supplementary material for: EZH2 expression is a prognostic biomarker in patients with colorectal cancer treated with anti-EGFR therapeutics
Source: Oncotarget. 2017 Jan 27;8(11):17810–8. doi: 10.18632/oncotarget.14863 (PMC5392288; doi:10.18632/oncotarget.14863)
Supplement: Supplementary file 1 [file oncotarget-08-17810-s001.pdf]

EZH2 expression is a prognostic biomarker in patients with colorectal cancer treated with anti-EGFR therapeutics

Supplementary Materials

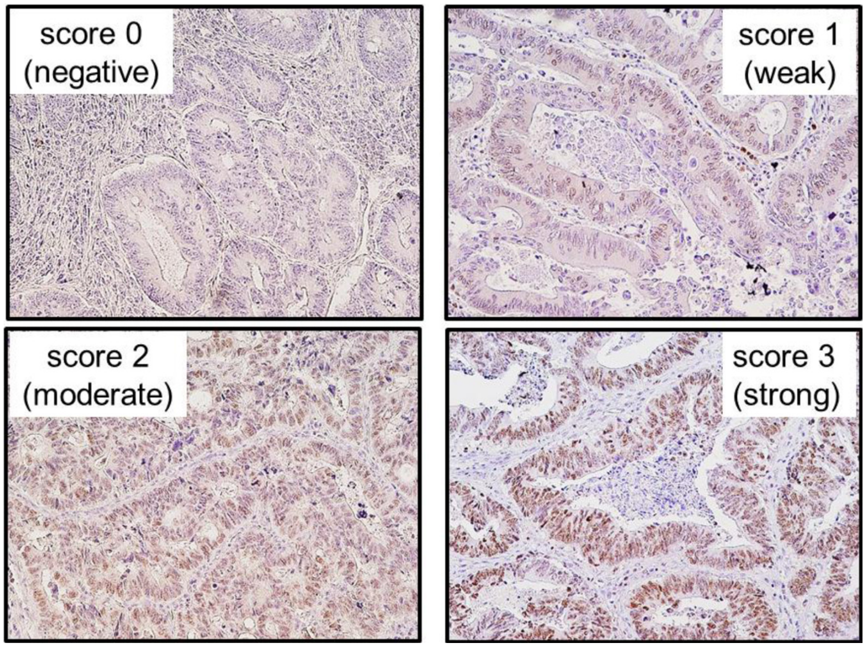

Supplementary Figure 1: Immunohistochemical findings related to EZH2 expression in colorectal cancers. Score 0 (negative), score 1 (weak), score 2 (moderate), and score 3 (strong) EZH2 expressions were observed in 11%, 21%, 18%, and 50% of the 109 colorectal cancer tissues, respectively. Five random high-power fields were evaluated per lesion to determine the mean nuclear positivity, which was categorized as follows: score 0 (negative, < 5%), score 1 (weak, 5%–39%), score 2 (moderate, 40%–79%), or score 3 (strong, ≥ 80%).

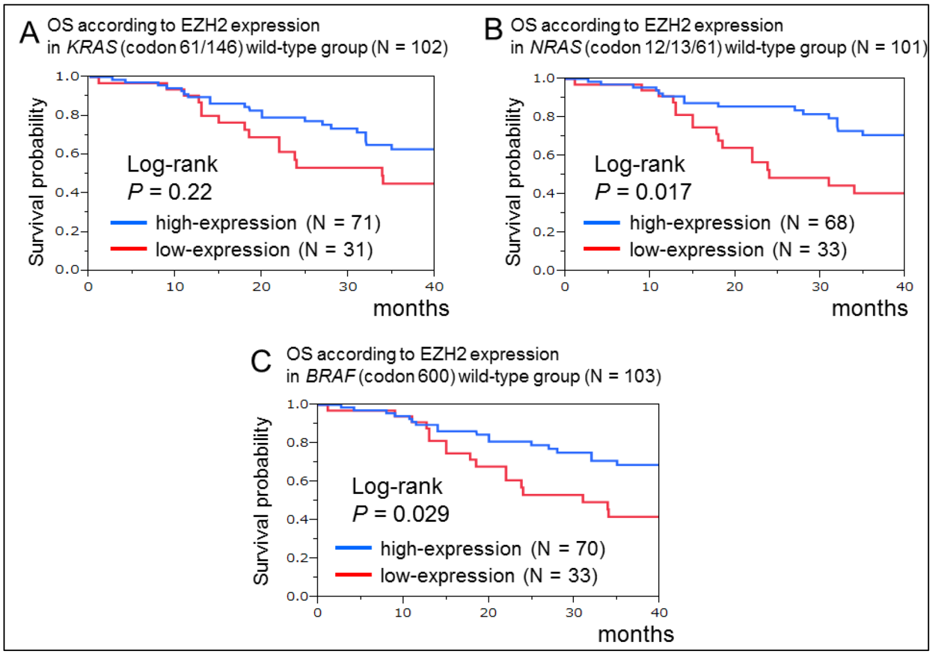

Supplementary Figure 2: Kaplan–Meier survival curves of patients treated with anti-EGFR therapeutics in *KRAS* (codon 61/146), *NRAS* (codon 12/13/61), and *BRAF* (codon 600) wild-type groups. (**A**) Overall survival according to EZH2 expression in the *KRAS* wild-type group (N = 102). (**B**) Overall survival according to EZH2 expression in the *NRAS* wild-type group (N = 101). (**C**) Overall survival according to EZH2 expression in the *BRAF* wild-type group (N = 103)
